# Change in adipose tissue characteristics and lipid metabolism in natural grazing Mongolian cattle with age

**DOI:** 10.5713/ab.24.0706

**Published:** 2025-02-27

**Authors:** Xueting Yu, Lu Chen, Xige He, Yunfei Han, Yajuan Huang, Rina Sha, Gerelt Borjigin

**Affiliations:** 1 College of Food Science and Engineering, Inner Mongolia Agricultural University, Hohhot, China; 2 State Key Laboratory of Reproductive Regulation and Breeding of Grassland Livestock, School of Life Sciences, Inner Mongolia University, Hohhot, China; 3 Inner Mongolia Academy of Agricultural and Animal Husbandry Sciences, Hohhot, China

**Keywords:** Lipids, Metabolite, Mongolian Cattle, Subcutaneous Fat

## Abstract

**Objective:**

This study aimed to elucidate the lipid composition, characteristics, and metabolic mechanisms of subcutaneous adipose tissue (SAT) in natural grazing Mongolian cattle during its development.

**Methods:**

The experimental animals consisted of castrated Mongolian cattle, divided into two groups: 10 months old (10 M, n = 12, average weight: 113.26±0.87 kg) and 34 months old (34 M, n = 12, average weight: 390.44±1.23 kg). All animals were selected from the same herd in the Eren Nur grasslands, Xilingol, Inner Mongolia, China. Browning-related genes in SAT were determined using quantitative reverse transcription polymerase chain reaction with 3 replicates for each cattle, and six animals from each group were randomly selected for non-targeted lipidomic techniques to analyze the changes in lipid metabolism with 6 replicates.

**Results:**

The yellowness value and relative expression of mitochondrial biogenesis-specific markers, thermogenic markers, uncoupling protein-2 (*UCP2*), and beige adipocyte marker genes in the SAT increased with age. Multivariate analysis revealed 172 significantly different lipids (SDLs). Triacylglycerols and phosphatidylcholines showed high relative contents. Glycerol and sterol lipid levels were predominantly upregulated during development, whereas phospholipids and sphingolipids were primarily upregulated at 10 M. Developmental time greatly influenced the fatty acid composition of neutral lipids, phospholipids, and free fatty acids. Pathway analysis demonstrated that the 172 SDLs were primarily involved in glycerophospholipid, glycerolipid, ether lipid, and sphingolipid metabolism.

**Conclusion:**

The developmental stage significantly affected the lipid quantity, composition, and metabolism of SAT in Mongolian cattle. Certain functional lipids, such as medium-long-chain triglycerides, fatty acid ester of hydroxyl fatty acid, and vitamin D, were detected in the yellow fat of natural grazing Mongolian cattle. These findings provide a comprehensive reference for the metabolic characteristics of yellow fat in Mongolian cattle, contributing to the meat industry chain.

## INTRODUCTION

Fat deposition in animals is closely associated with production efficiency and meat quality, flavor, and juiciness, as well as human health and nutrition. The two main types of adipocytes in mammals are white adipocytes, which store energy in white adipose tissue (WAT), and brown adipocytes responsible for heat production (thermogenesis) in brown adipose tissue (BAT). Furthermore, brown adipocytes within BAT harbor uncoupling protein-1 (*UCP1*)-rich mitochondria, indicating that BAT specializes in energy expenditure rather than energy storage. In addition to white and brown adipocytes, a third type called beige adipocytes have recently been observed within WAT depots as inducible thermogenic cells that exhibit mixed functions of energy storage and expenditure [1]. They have garnered significant interest recently owing to their ability to regulate energy balance and improve metabolic health [2]. Long-term cold stimulation, sympathetic nerve stimulation, peroxisome proliferator-activated receptor γ agonists, and exercise have been reported to induce the formation of beige adipocytes [3], a phenomenon known as “browning”. In cattle, cold stimulation has been found to result in browning of subcutaneous WAT, which is more susceptible to cold exposure owing to its location in the body [4].

The backfat of cattle is an important economic trait due to its strong implications for meat yield, meat flavor, and juiciness [5], indicating that backfat deposition plays an important role in beef production. Du et al [6] analyzed subcutaneous, visceral, and abdominal adipose tissues (SAT, VAT, and AAT) in Huaxi cattle. They found that triacylglycerol (TGs) content in SAT was significantly higher than in VAT and AAT, while diacylglycerols (DGs) and lysophosphatidylcholines (LPCs) levels were significantly lower. In addition, Xiong et al [7] found that graze-feeding cattle had a higher proportion of DG in SAT but lower proportions of phosphatidylethanolamines (PEs), sphingomyelins (SMs), and TGs than stall-feeding cattle. Liu et al [8] found that adipose tissue of Lilu yellow cattle energy metabolism was active from 12 to 24 months but inhibited at 30 months. Marcher et al [9] demonstrated that cold exposure can increase the levels of long-chain TG species in adipose tissues, thereby increasing heat production. These results suggested that lipid metabolism in the adipose tissue of cattle depends on different parts, feeding conditions, age, and cold climate. The SAT color in cattle is an essential component of beef carcass quality. In our previous study, we compared the adipose tissue of grazing and house-breeding cattle and identified 456 differentially expressed genes and 66 differentially expressed miRNAs [10]. Further research indicated that the redness (a^*^) and yellowness (b^*^) values were significantly higher in grazing cattle than in house-breeding cattle (in submit). In addition, Walsh et al [11] provided five different diets to steers and observed that the yellowness of carcass fat generally decreased in the following sequence: grass silage>whole crop wheat silage>ad libitum concentrates>maize silage>alkalage. The slaughter season also affected the color parameters of cattle, Węglarz et al [12] observed that color parameters in the tissues of slaughtered cattle were higher in winter than in summer (The summary table of comparative literature findings is shown in Supplement 1).

Mongolian cattle (*Bos taurus*) are the most representative indigenous superior breed. They naturally graze in an environment with abundant forage species throughout the year and are characterized by their phenotypic feature of yellow fat, which becomes more yellow with age. Because of the eating habits of the local people, yellow fat in natural grazing cattle is considered edible and plays a role in human health and nutrition. For example, oil extracted from fat is used as an alternative to edible vegetable oils used in fermented and blood sausages, and other foods. Natural grazing cattle are typically born in February and slaughtered in December. The distinct growth cycles of cattle around seasonal variations may affect fat characteristics. However, the lipid composition, characteristics, and metabolism of SAT in natural grazing Mongolian cattle at different growth stages remain largely unclear. Thus, in the present study, we analyzed the fat color, relative expression of genes associated with browning, and lipid metabolite profiles in grazing Mongolian cattle 10 months old (10 M) and 34 months old (34 M). The purpose of this study was to investigate the metabolism mechanism of yellow fat formation during the growth of Mongolian cattle. Additionally, this study will provide theoretical data for future research on the formation mechanism of yellow fat, explore its potential functions, and enhance the utilization of yellow fat.

## MATERIALS AND METHODS

### Animal care

This study was reviewed and approved by the Ethics Committee of Inner Mongolia Agricultural University (license no. IMAU-2022086).

### Sample collection

The experimental animals included 24 castrated Mongolian cattle (*Bos taurus*) into two groups: 10 M (n = 12, average weight: 113.26±0.87 kg) and 34 M (n = 12, average weight: 390.44±1.23 kg). All the animals were selected from the same herd in the Eren Nur grasslands of Xilingol, Inner Mongolia, China (111°54′72.1″ E, 43°35′18″ N and 900 to 1400 m asl), where the forage species are abundant. This region is a typical steppe dominated by a continental temperate climate, with a total annual rainfall of 177 mm and an annual average temperature of 4.3°C. The extreme maximum summer and minimum winter temperatures were recorded as approximately 35°C and −40°C, respectively. Following the local traditional grazing management protocol, animals are accustomed to voluntary movement and free feeding (also called natural grazing) throughout their lifespan. Samples were collected in December 2022 (Slaughter time). After overnight fasting, all animals used in this study were transported to a commercial abattoir, where they were immediately slaughtered by professionals following standard commercial procedures. After slaughter, 70 g SAT (backfat at the 12th and 13th ribs) was collected from each sample. 20 g were taken from each sample stored in marked cryogenic tubes, and rapidly snap-frozen in liquid nitrogen for subsequent measurement of relative mRNA expression levels and lipidomic analysis. Simultaneously, 50 g of adipose tissue from each sample was packed in sterile polyethylene-sealed bags, stored in a cooler, and then frozen at −20°C for subsequent analysis of adipose tissue color.

### Adipose tissue color measurement

A WSC-S automatic colorimeter (Konica Minolta Inc, Shanghai, China) was used to measure the SAT color of each sample. The color was recorded in triplicate for each sample using three indexes: L* (lightness), a* (redness), and b* (yellowness). The colorimeter was equipped with an Φ20 mm measuring area and 0/d illumination angle (CIE standard illuminant D65), and calibrated with a white standard plate before starting each measurement as per the directions of the instrument manual.

### Total RNA extraction, complementary DNA synthesis, and quantitative reverse transcription polymerase chain reaction

Total RNA was extracted from 100 mg of adipose tissue using the TRNzol Universal Reagent (TIANGEN, Beijing, China). Complementary DNA (cDNA) synthesis was performed using the HiScript II Q RT SuperMix (Vazyme, Nanjing, China), according to the manufacturer’s instructions. Quantitative reverse transcription polymerase chain reaction (RT-qPCR) was performed to characterize the relative expression of target genes using specific primers. The qPCR mixture (20 μL) included 10 μL of 2 × ChamQ Universal SYBR qPCR MASTER Mix (Vazyme), 0.4 μL of forward and reverse primer each (10 μM each), 8.2 μL of nuclease-free water, and 1.0 μL of cDNA. qPCR was conducted using the LightCycler 96 real-time PCR system (Roche, Basel, Switzerland), with adjustments made according to the procedure of Yang et al [13]. The procedure included a preincubation at 95°C for 30 s, followed by 45 cycles of denaturation at 95°C for 5 s, and annealing and extension at 60°C for 30 s. After the PCR cycles, a melting curve was generated (95°C for 5 s, 60°C for 60 s, and 95°C for 1 s [continuous]) to discriminate between the specific amplicons and nonspecific amplification products. Each sample was measured in triplicate. Results were calculated using the 2^−ΔΔCt^ method, normalized by genomic glyceraldehyde-3-phosphate dehydrogenase (GAPDH). According to the mRNA information of NADH dehydrogenase subunit 1 (ND1), mitochondrial biogenesis-specific markers, thermogenic markers, *UCP2*, and beige adipocyte marker genes registered on the NCBI website, the primers used were designed with Primer Premier 5 software (Premier Biosoft International, Palo Alto, CA, USA; Supplement 2). Primers were synthesized by Sangon Biotech (Shanghai, China).

### Measurement of mitochondrial DNA copy number

For analyzing the mitochondrial DNA (mtDNA) copy number, genomic DNA was extracted from adipose tissue using a DNA extraction kit (TIANGEN). mtDNA levels were determined by calculating the ratio of ND1 to GAPDH using RT-qPCR. The relative mtDNA copy number was assessed using the 2^−ΔCT^ method (ΔCT = CT_ND1_ − CT_GAPDH_).

### Lipid extraction and lipidomic analysis

Lipid metabolite extraction and lipidomic analysis were performed by LC-Bio Technologie Co., ltd (Hangzhou, China). The samples were thawed on ice after removing from the −80°C freezer. Lipids were subsequently extracted using a 50% methanol buffer. Specifically, 120 μL of precooled 50% methanol buffer was added to the mixture of the metabolites, which was then vortexed for 1 min and incubated for 10 min at 25°C. The resulting extraction mixture was stored at −20°C overnight. The mixture was centrifugated at 4,000×g for 20 min and stored at −80°C before analysis. Additionally, 10 μL of each extraction mixture was removed to prepare pooled quality control (QC) samples.

All lipid samples were analyzed using liquid chromatography-mass spectrometry (LC-MS) by an ultra-high-performance liquid chromatography (UPLC) system (ExionLC; SCIEX, Framingham, UK) and a high-resolution tandem mass spectrometer (TripleTOF 5600+; SCIEX). The analytical conditions were as follows: column ACQUITY UPLC HSS T3 (1.8 μm, 2.1×100 mm, Waters, Wilmslow, UK) and solvent system A (water, 0.1% formic acid) and B (acetonitrile, 0.1% formic acid). The gradient elution conditions were as follows with a flow rate of 0.4 mL/min: 5% solvent B for 0 to 0.5 min, 5% to 100% solvent B for 0.5 to 7 min, 100% solvent B for 7 to 8 min, 100%–5% solvent B for 8 to 8.1 min, and 5% solvent B for 8.1–10 min. The column temperature was maintained at 35°C. The TripleTOF 5600+ system was used to detect the metabolites eluted from the column. The quadrupole time-of-flight (TOF) was operated in positive and negative ion modes. The curtain gas pressure was set to 30 psi, and the ion source gas 1 and gas 2 pressures were set to 60 psi. The interface heater temperature was 650°C, and the ion spray voltages were 5,000 (positive) and −4,500 V (negative). The MS data were acquired in the information dependent acquisition mode. The TOF mass range was 60 to 1,200 Da. Dynamic exclusion was implemented.

Mass accuracy was calibrated for every 20 samples during the acquisition period. Additionally, QC samples were included in every 10 detection and analysis samples to assess the repeatability of the analysis process. Variations in the QC results were used to correct systematic errors throughout the batch experiments.

### Statistical and bioinformatics analyses

The normality of data distribution was tested using the Shapiro-Wilk test. SPSS 18.0 (SPSS Inc., Chicago, IL, USA) was used for Pearson’s correlation analysis and one-way analysis of variance (ANOVA) to compare the color parameters and gene mRNA expression data. Unless stated otherwise, the results are presented as the mean±standard error of the mean. The statistical level of significance was set at * p<0.05 and ** p<0.01, and a correlation coefficient >0.8 or <−0.8 indicated a strong correlation.

The LC-MS raw data were analyzed using the XCMS software (SCIEX, Warrington, UK), with retention time and M/Z data utilized for ion identification. Subsequently, the online Kyoto Encyclopedia of Genes and Genomes (KEGG), Human Metabolome Database (HMDB), and in-house databases were employed for level-one and level-two identification and annotation. Before MS2 analysis, all samples underwent data normalization using the probabilistic quotient normalization algorithm. Subsequently, QC-robust spline batch correction was performed using the QC samples. The p*-*value was analyzed using Student’s *t*-tests and then adjusted for multiple tests using a false discovery rate (Benjamini-Hochberg). Principal component analysis (PCA) and partial least squares discriminant analysis (PLS-DA) were performed using OmicStudio (https://www.omicstudio.cn/tool). Significantly different lipids (SDLs) were screened using the following criteria: p*-*value (p<0.05), fold-change (FC≥2 or FC≤0.5), and variable importance in projection (VIP>1). The KEGG compound database and the MetaboAnalyst 5.0 platform were used to map and differentiate lipid-related metabolic pathways. Topological analyses were performed on these SDLs to calculate the p-values and pathway effects. Significant pathways were identified based on p*-*value<0.05 and impact value>0.1. Subsequently, the umapped metabolic pathways were subjected to metabolite set enrichment analysis.

## RESULTS AND DISCUSSION

### Adipose tissue color analysis

**Figure 1:**
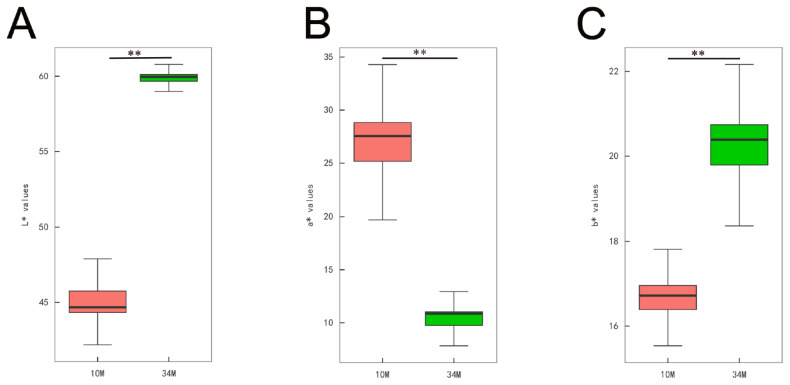
Subcutaneous adipose tissue (SAT) color between 10 (10 M) and 34 months old (34 M) Mongolian cattle. (A) L* (lightness) values. (B) a* (redness) values. (C) b* (yellowness) values. ** indicates an extremely significant difference between different ages for SAT (p<0.01).

SAT color in cattle is a crucial indicator of beef carcass quality. As shown in Figure 1, the L* and b* values of SAT were significantly higher in the 34 M group than in the 10 M group (p<0.01). In contrast, the a* value of 10 M cattle was higher than that of 34 M cattle (p<0.01). Węglarz et al [12] observed that color parameters in the tissues of slaughtered cattle were higher in winter than in summer. The higher L* value in 34 M may be due to a lower concentration of natural antioxidants, resulting in lipid oxidation, discoloration, and water loss. Sargentini et al [14] reported a significant increase in both L* and b* values with the age of Maremmana cattle (p<0.05), which was consistent with our findings. The a* value is affected by myoglobin content and oxidation state. Myoglobin concentration in cattle increases until 24 months of age [15]. Therefore, the decrease in a* value observed for 34 M may be attributed to a decline in myoglobin content. Yellowness is considered the most crucial color parameter of adipose tissue. It has been well documented that natural grazing promotes carotenoid accumulation, leading to simultaneous yellow fat formation. In addition, older cattle tend to have more yellow carcass fat [16]. Thus, these color differences could be partly attributed to climate change, the age of the animals, and their diets.

### Expression of mtDNA copy numbers, mitochondrial biogenesis-specific markers, and browning-related factors

**Figure 2:**
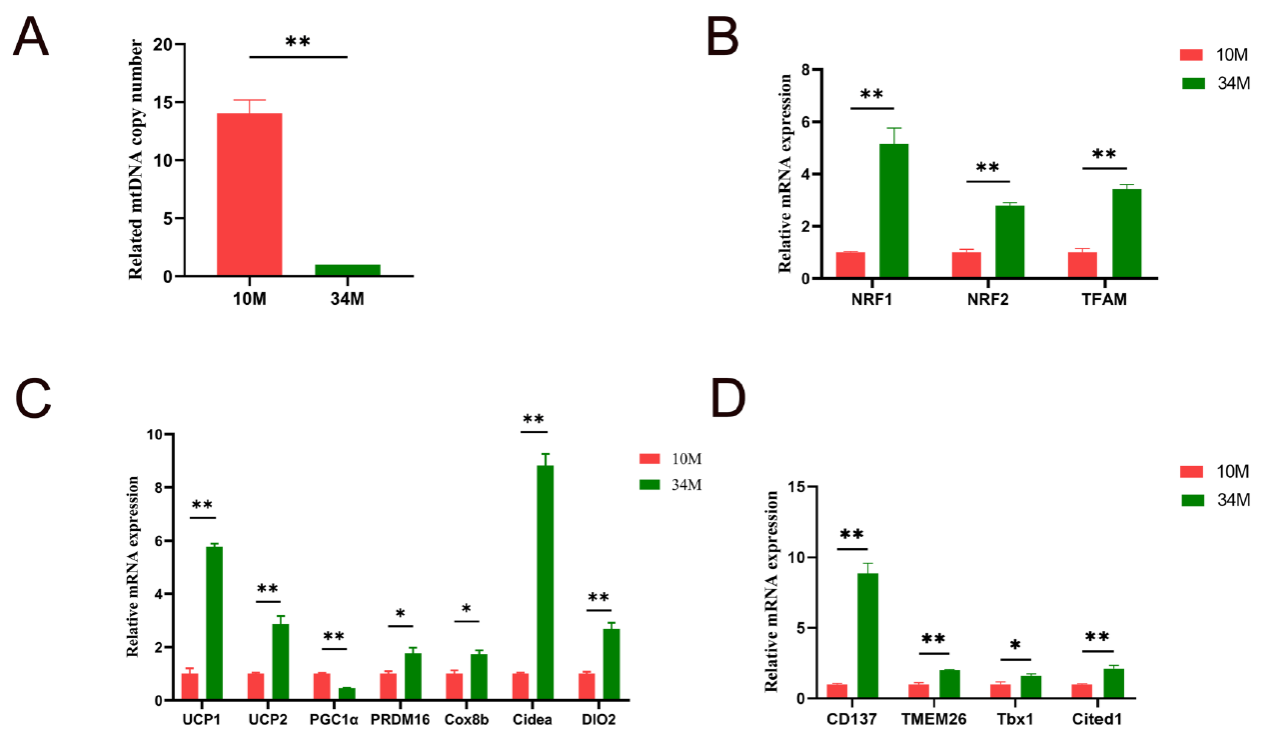
Expression of browning-related factor. (A) Relative mitochondrial DNA (mtDNA) copy number. (B) Relative mRNA expression levels of mitochondrial biomarkers. (C) Brown/beige adipocyte-related and uncoupling protein-2 (*UCP2*) mRNA-relative expression levels. (D) Relative expression levels of beige-fat-selective marker genes. Data are presented as the mean±standard error of the mean. * indicates significant differences between different ages for SAT (p<0.05); ** indicates extremely significant difference (p<0.01). SAT, subcutaneous adipose tissue.

The role of beige adipocytes in energy regulation and metabolism has been studied extensively. The key features of browning include an increase in mtDNA copy number, upregulation of marker genes specific to mitochondrial biogenesis, and enhanced expression of browning-related genes [4]. As shown in Figure 2A, the relative mtDNA copy number in the SAT of 34 M cattle was significantly reduced (14.0-fold; p<0.01). In general, the number of mtDNA copies, which exhibits a significant positive correlation with lipogenesis, increases substantially during the first year of an animal’s life [15]. Thus, the mtDNA copy number declines with advancing age. Our result was consistent with that reported by Barazzoni et al [17]. However, the relative mRNA expression levels of critical regulators of mitochondrial biogenesis, including nuclear respiratory factor 1 (*NRF1*), *NRF2*, and mitochondrial transcription factor A (*TFAM*), were markedly increased in the SAT of 34 M cattle (5.2-, 2.8-, and 3.4-fold, respectively; p<0.01; Figure 2B). Hou et al [18] demonstrated that mitochondrial biogenesis increases during browning. Thus, aged Mongolian cattle enhance mitochondrial biogenesis to stimulate browning, possibly due to long-term grazing or cold climate.

Figure 2C shows the relative expression levels of brown/beige adipocyte-related adaptive thermogenesis genes and *UCP2* in the SAT of 34 M cattle compared with 10 M cattle. The qPCR results indicated that the relative expression levels of mRNAs for *UCP1*, *UCP2*, PR domain-containing (*PRDM16*), cytochrome c oxidase subunit 8B (*Cox8b*), cell death-inducing DFFA-like effector A (*Cidea*), and deiodinase, iodothyronine, type II (*DIO2*) were significantly increased in the 34 M group (5.8-, 2.9-, 1.8-, 1.7-, 8.8-, and 2.7-fold, respectively, p<0.05). In particular, high *UCP1* expression in mitochondria is the most striking feature of brown and activated beige adipocytes. *UCP1* is essential for maintaining energy homeostasis and is closely associated with energy metabolism. Similarly, *Cidea* is involved in the regulation of energy homeostasis and lipid metabolism [19]. *PRDM16* is a critical transcriptional regulator of brown and beige fat and is primarily involved in the upregulation of BAT/beige adipocyte functions and heat production [20]. In contrast, the expression of peroxisome proliferator-activated receptor gamma coactivator alpha (*PGC1α*) was downregulated in the 34 M group (2.2-fold, p<0.05). This downregulation may be attributed to various repressors that target *PGC1α*, inhibiting beige adipocyte development [21]. Furthermore, the level of *PGC1α*, is positively correlated with mtDNA amount and negatively correlated with aging [22]. The observed changes in relative gene expression in the present study were consistent with the findings of previous studies, providing further support for the rationale behind the experimental group design. Therefore, we further examined the expression of beige adipocyte markers.

As shown in Figure 2D, the relative mRNA expression levels of cluster of differentiation 137 (*CD137*), transmembrane protein 26 (*Tmem26*), T-box transcription factor 1 (*Tbx1*), and Cbp/p300 interacting transactivator with Glu/Asp rich carboxy-terminal domain 1 (*Cited1*) were markedly upregulated in the SAT of 34 M cattle (8.9-, 2.0-, 1.6-, and 2.1-fold, respectively; p<0.05). Beige adipocytes can be distinguished from brown and white adipocytes through *CD137*, *Tmem26*, *Tbx1*, and *Cited1* expressions. The expression of these genes may be affected by feeding and seasonal changes. Asano et al [23] found significantly higher *UCP1* expression in cattle’s adipose tissue in the concentrate diet group than in the roughage diet group. However, cold exposure enhances the gene expression of beige adipocyte, thermoregulatory, and mitochondrial biogenesis markers in cattle adipose tissue compared to a high-energy diet [24]. Overall, the abundance of beige adipocytes may be higher in aged cattle than in calves, potentially contributing to the observed SAT yellowing in aged cattle. The findings of this study contradict previous observations in mice and humans [2]. Although species and tissue differences can partially explain the variations in beige adipocytes with age, the primary factors contributing to these disparities are likely the cold climate and year-round free movement of Mongolian cattle. Studies have shown that mice housed under cold conditions undergo marked remodeling of their WAT, characterized by an accumulation of beige adipocytes [3]. Additionally, in the inguinal WAT of long-term exercise-aged mice, the expression of genes related to mitochondrial biogenesis, thermogenesis, and beige adipocytes was increased [25]. These findings suggest that the beige adipocyte markers, thermoregulatory markers, and mitochondrial biogenesis markers were upregulated in the SAT of 34 M cattle (p<0.05). This difference may be related to age, grazing behavior, feeding conditions, and seasonal changes. The increased expression level of these genes promotes lipid metabolism, maintains body temperature, and acclimatizes the cattle to the cold environment of the Mongolian Plateau.

### Correlation analysis of *UCP2* and brown/beige adipocyte-related adaptive thermogenesis genes

Table 1 shows the correlation analysis between *UCP2* and brown/beige adipocyte-related adaptive thermogenesis genes. The strongest correlation was observed between *UCP2* and *DIO2* (r = 0.999, p<0.01), closely followed by *Cidea* (r = 0.995, p<0.01) and *UCP1* (r = 0.992, p<0.01), implying the potential role of *UCP2* in energy metabolism during the growth of Mongolian cattle. These results are similar to those reported by Shigematsu et al [26], who found that *UCP2* expression levels were correlated with certain brown or beige adipocyte-related genes. Thus, the increased relative expression levels of *UCP2* may be related to the formation of beige adipocytes, although the precise mechanism remains unclear.

### Lipid metabolomics analysis

#### Overview of lipid metabolomics results

**Figure 3:**
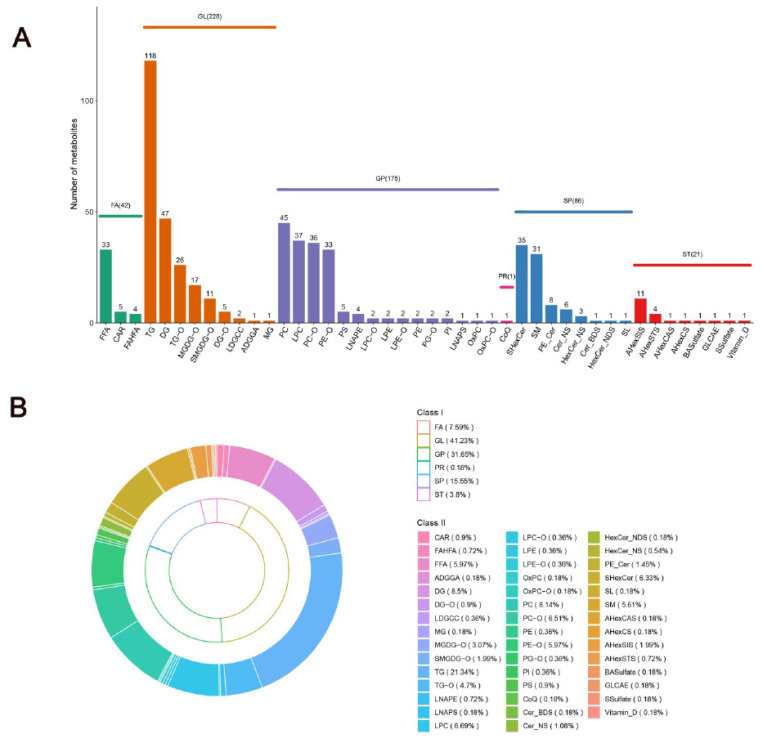
SAT from Mongolian cattle at different growth stages. (A) Quantities of lipid categories and subclasses. (B) Percentages of lipid categories and subclasses. GL, glycerolipids; FA, fatty acyls; GP, glycerophospholipids; SP, sphingolipids; ST, sterol lipids; PR, prenol lipids; FFA, free fatty acid; CAR, acylcarnitine; FAHFA, fatty acid ester of hydroxyl fatty acid; TG, triglyceride; DG, diacylglycerol; TG-O, ether-linked triacylglycerol; MGDG-O, ether-linked monogalactosyldiacylglycerol; SMGDG-O, semino lipid; DG-O, ether-linked diacylglycerol; LDGCC, lysodiacylglyceryl-3-O-carboxyhydroxymethylcholin; ADGGA, acyl diacylglyceryl glucuronid; MG, monoacylglycerol; PC, phosphatidylcholines; LPC, lysophosphatidylcholine; PC-O, ether-linked phosphatidylcholine; PE-O, ether-linked phosphatidylethanolamine; PS, phosphatidylserine; LNAPE, n-acyl-lysophosphatidylethanolamine; LPC-O, ether-linked lysophosphatidylcholine; LPE, lysophosphatidylethanolamine; LPE-O, ether-linked lysophosphatidylethanolamine; PE, phosphatidylethanolamine; PG-O, ether-linked phosphatidylglycerol; PI, phosphatidylinositol; LNAPS, n-acyl-lysophosphatidylserine; OxPC, oxidized phosphatidylcholine; OxPC-O, ether-linked oxidized phosphatidylcholine; CoQ, coenzyme Q; SHexCer, sulfatide; SM, sphingomyelin; PE_Cer, ceramide phosphoethanolamine; Cer_NS, ceramide non-hydroxyfatty acid-sphingosine; Cer_BDS, ceramide beta-hydroxy fatty acid-dihydrosphingosine; HexCer_NDS, hexosylceramide non-hydroxyfatty acid-sphingosine; SL, sulfonolipid; AHexSIS, acylhexosyl sitosterol; AHexSTS, acylhexosyl stigmasterol; AHexCAS, acylhexosyl campesterol; AHexCS, acylhexosyl cholesterol; BASulfate, bile acid sulfate; GLCAE, esterified glycolithocholic acid; SSulfate, sterol sulfate.

Lipid molecules, which are critical components of meat and vital functional substances in the body, initiate a cascade of physiological and biochemical processes. The lipid profiles of SAT in Mongolian cattle were examined and classified in this study. After filtering out the lipids detected in the positive and negative ion modes, 553 lipids were successfully characterized in the 10 and 34 M samples. These lipid molecules were further divided into six classes: glycerolipids (GLs; n = 228; 41.23%), glycerophospholipids (GPs; n = 175; 31.65%), sphingolipids (n = 86; 15.55%), fatty acyls (FAs; n = 42; 7.59%), sterol lipids (STs; n = 21; 3.80%), and prenol lipids (PRs; n = 1; 0.18%) (Figures 3A, 3B). These data suggest that GLs were the most abundant lipids. Among GLs, TGs were the most abundant lipid molecules, accounting for 21.34% of the total lipids, followed by DGs, which accounted for 8.5% of the total lipid molecules (Figure 3B). Furthermore, the lipid molecules proportions of four key subclasses of GPs were analyzed, including phosphatidylcholines (PCs), LPCs, ether-linked phosphatidylcholine (PC-O), and ether-linked phosphatidylethanolamine (PE-O). The percentage of their composition was 8.14%, 6.69%, 6.51%, and 5.97%, respectively (Figure 3B). Sphingolipids mainly existed in the form of sulfatide (SHexCer) and SM, accounting for 6.33% and 5.61% of the total lipid molecules, respectively (Figure 3B). Our lipidomics results were consistent with those of Xiong et al [7], who reported that the lipids in yak SAT were mainly composed of DGs, LPCs, PCs, PEs, SMs, and TGs. These results indicate the diversity and complexity of SAT lipids in Mongolian cattle.

#### Identification of characteristic lipids

**Figure 4:**
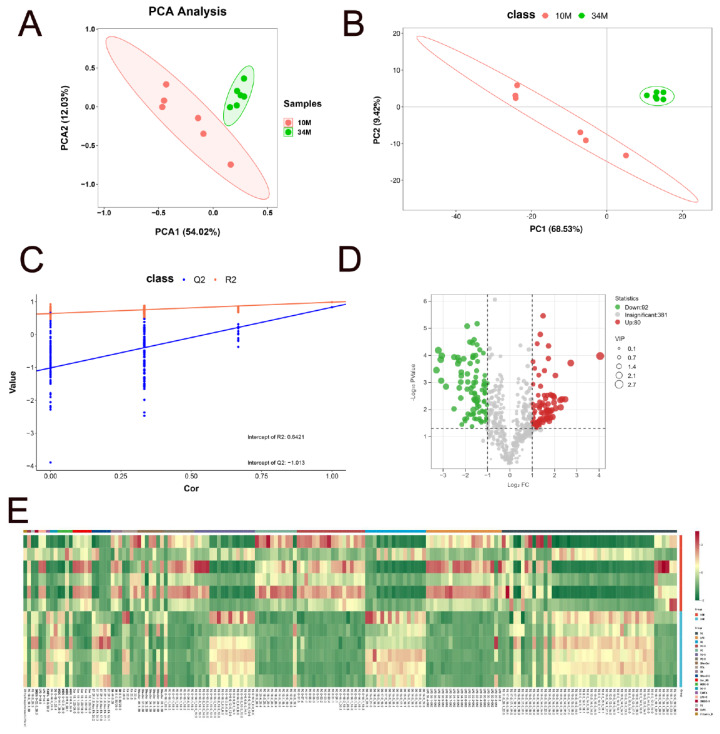
Differences in the SAT of 10 and 34 M Mongolian cattle. (A) Principal component analysis (PCA) scatter plot of differentially expressed lipid metabolites. (B) Based on the extracted spectral data, partial least squares discriminant analysis (PLS-DA) score plots of the SAT from Mongolian cattle. (C) PLS-DA permutation plot. (D) Volcano map of differential lipids. Under the triple screening conditions of VIP+FC+p-value, the abscissa represents the fold-change (log2FoldChange), the vertical axis indicates the significance level of the difference (log10 p-value), and the size of the dot represents the VIP value. Green bubbles indicate high expression in the SAT of 10 M and red bubbles indicate high expression in the SAT of 34 M. (E) Heatmap analysis of 172 significantly different lipids (SDLs) in 10 and 34 M groups. SAT, subcutaneous adipose tissue; VIP, variable importance in projection; FC, fold change.

The lipid profiles of SAT in Mongolian cattle at 10 and 34 M were compared using PCA and PLS-DA methods to identify differential lipid molecules. The PCA score plot demonstrated grouping by principal components PC1 and PC2, which explained 54.02% and 12.03% of the total variation, respectively (Figure 4A). Remarkably, PCA showed discrimination between the two groups, suggesting a difference in lipid composition between the 10 and 34 M groups. PLS-DA (Figures 4B, 4C), a supervised dimensionality reduction method that incorporates class labels into the analysis, maximizes the separation between different predefined groups (e.g., healthy vs. diseased) in the dataset. The model was validated with a permutation test to ensure that the PLS-DA model’s discrimination was not due to data overfitting. The permutation test (200 repeats) showed an R^2^ value of 0.6421 and a Q^2^ value below 0, suggesting a well-fitted model. The lipid metabolites were further filtered using a combination of p<0.05 coupled with FC≥2 or FC≤0.5 and VIP>1 predicted by a PLS-DA model to identify representative lipid metabolites. In total, 172 SDLs were identified in the volcano plot (Figure 4D). For better visualization and analysis, a heatmap was generated to compare the SDLs of each lipid subclass (Figure 4E). 172 SDLs were filtered with p<0.05, FC≥2 or FC≤0.5, and VIP>1, and showed an upward/downward trend in Supplement 3. The levels of 92 lipids in the SAT of 34 M cattle were considerably lower than those in the SAT of 10 M cattle, including 15 TGs, 19 LPCs, 18 PC-Os, 10 PCs, 4 ether-linked triacylglycerols (TG-Os), 7 PE-Os, 5 SHexCers, 2 free fatty acids (FFAs), 3 SMs, 5 ceramide non-hydroxy fatty acid-sphingosines (Cer_NSs), 1 ether-linked monogalactosyldiacylglycerol (MGDG-O), 2 LPCs, and 1 oxidized phosphatidylcholine (OxPC). The remaining 80 lipids exhibited an increase in the SAT of 34 M cattle, including 31 TGs, 1 LPC, 16 DGs, 1 PC, 12 TG-Os, 1 PE-O, 2 SHexCers, 2 FFAs, 5 acylhexosyl sitosterols (AHexSISs), 3 MGDG-Os, 2 ether-linked diacylglycerols (DG-Os), 1 fatty acid ester of hydroxyl fatty acid (FAHFA), 1 seminar lipid (SMGDG-O), 1 phosphatidylserine (PS), and 1 vitamin D.

Based on the heatmap observations, GLs were the main components of cattle lipids. The TG and DG contents in the SAT gradually increased with age. An increased deposition of phospholipid molecules (LPC, PC-O, PC, and PE-O) in the SAT of 10 M cattle. These results were consistent with the findings of Li et al [27]. The total lipid content during development likely influences the balance between neutral lipids and phospholipids. Therefore, if the total lipid content of cattle increases, neutral lipids may account for a more significant proportion, with a corresponding decrease in the phospholipid content. Phospholipids are essential components of cell membranes, and their amount remains relatively constant or slightly increases as fat content increases [28].

Ether phospholipids (PC-O, PE-O, and LPC-O) were primarily upregulated at 10 M. Most ether phospholipid species are plasmalogens. In mammalian cells, plasmalogens contain a vinyl ether-linked fatty alcohol at the sn-1 position of the glycerol backbone and an acyl-linked fatty acid at the sn-2 position. Typically, the sn-1 positions are occupied by either 16- or 18-carbon ether or vinyl ether moieties, whereas the sn-2-linked acyl chain is a polyunsaturated fatty acid (PUFA). Our findings align with those of Maeba et al [29], who indicated that plasmalogen content generally decreases in aged mammals. Additionally, plasmalogens have been implicated in lipid droplet formation, and an increase in lipid droplet biogenesis leads to endoplasmic reticulum (ER) stress. The primary mechanisms that drive this association are a combination of reduced phospholipid synthesis and upregulated TG synthesis [30]. Therefore, it can be inferred that the decrease in plasmalogens in subcutaneous fat in aged Mongolian cattle may be due to ER stress.

Sphingolipids consist of a sphingoid base that is N-linked to a FA chain. In this study, SM and Cer_NS were upregulated at 10 M. Alexaki et al [31] indicated that de novo sphingolipid biosynthesis is required for adipocyte survival and metabolic function. The de novo synthesis pathway begins with the condensation of serine and palmitoyl coenzyme A (CoA) to form ceramide (Cer). Cer can be further metabolized to SM and Cer_NS. We suggest that there may be higher levels of palmitoyl CoA and serine in SAT at 10 M than at 34 M. Chaurasia et al [32] also demonstrated that palmitoyl CoA and serine are key determinants of elevated Cer levels.

In the present study, ST species, including AHexSIS and vitamin D, were abundant in the SAT of 34 M cattle. The top five SDLs with the highest VIP values were observed in both groups (Supplement 4). ST (29:1; 0; Hex; FA 17:1) and ST (29:1; 0; Hex; FA 17:0), which are considered potential biomarkers to distinguish yellow fat, were upregulated in the SAT of 34 M cattle. Besides, Bonnet et al [33] emphasized that adipose tissue is a primary reservoir of vitamin D and its metabolites, mainly in the form of cholecalciferol and 25(OH)D3. Vitamin D is a multifunctional hormone (steroid) and is among the most important biomolecules for regulating and promoting sustainable health.

FFA (22:0) and FFA (28:0) were upregulated in the SAT of 34 M cattle, whereas FFA (22:6) and FFA (22:5) were upregulated in the SAT of 10 M cattle. Wood et al [28] demonstrated that an increase in saturated fatty acid (SFA) content is related to the acquisition of total ruminal functionality in cattle compared with calves. Therefore, increased ruminal activity with increasing ruminant age could explain the increase in SFA observed with increasing slaughter age. The essential health benefits of docosapentaenoic acid (DPA; 22:5n–3) and docosahexaenoic acid (22:6n–3) have been extensively studied. These fatty acids play essential roles in the optimal functioning and maintenance of health and well-being throughout human life. Interestingly, a certain intensity of FAHFA was upregulated in the SAT of 34 M cattle, which was detected in both caribou and moose meat [34]. FAHFAs are considered to be functional lipids and have been shown to improve insulin sensitivity, increase insulin secretion from the pancreas, and attenuate inflammation within adipose tissue in mice [35].

In summary, compared to the 10 M group, the expression of TG, DG, and STs was upregulated in the 34 M group, while phospholipids and sphingolipids were downregulated. Xiong et al [7] reported that the stall-feeding cattle had higher levels of PEs, SMs, and TGs compared to grazing cattle, but lower levels of DGs. This difference may be attributed to age and feeding conditions. Additionally, vitamin D and FAHFA functional lipids were discovered for the first time in the yellow fat of natural grazing cattle. The variation of yellow fat lipid metabolism during the growth of natural grazing Mongolian cattle revealed its functional compositions and promoted its utilization.

#### Distribution and composition of fatty acids in lipid molecules

**Figure 5:**
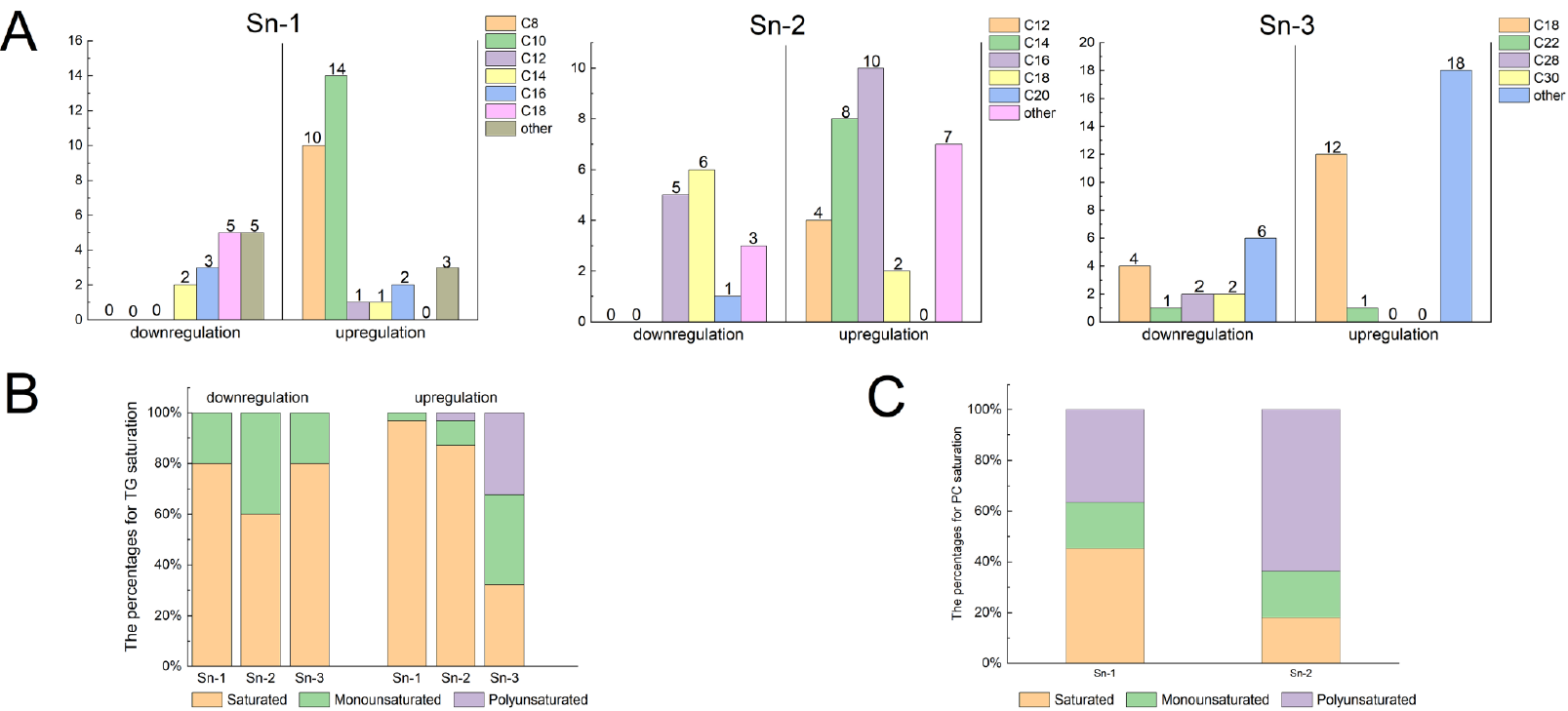
Distribution of different sites and fatty acid composition in TG and PC. (A) Changes in the side chain fatty acid length of 67 differential TG molecules. (B) Saturation degree of side chain fatty acids in TG molecules. (C) Saturation degree of side chain fatty acids in 11 differential PC molecules. Upregulation indicates high lipid abundance in the SAT of 34 M Mongolian cattle, while downregulation indicates high lipid levels in the SAT of 10 M Mongolian cattle. TG, triacylglycerol; PC, phosphatidylcholines; SAT, subcutaneous adipose tissue.

Most FA molecules participate in the formation of glycerol backbones through esterification, thereby generating GP and GL. These molecules interact with other complex lipids, influencing various aspects such as flavor compound formation and nutritional value. As shown in Figure 5A, an increase in medium-chain fatty acids (MCFAs) at the sn-1 site of TG molecules, primarily consisting of C8 and C10, was observed in 34 M cattle. Conversely, the FA composition at the sn-1 site of the TG molecules primarily consisted of long-chain fatty acids (LCFAs) in 10 M cattle. Additionally, ultra-LCFAs were found at the sn-3 sites of TG. We suggest that this may be due to the greater sensitivity of calves to cold. Cold stimulation can lead to highly selective increases in the abundance of long-chain, ultra-long-chain, and odd-chain acyls [9]. Additionally, most of the TGs upregulated in the SAT of 34 M cattle consisted of MCFAs and LCFAs. In contrast, those with downregulated expression had fewer short-chain fatty acids and MCFAs. Long-chain TGs, mainly obtained through daily diet, have a slow metabolic rate, and their long-term consumption may lead to fat accumulation in the body. However, medium- and long-chain triglycerides (MLCT), which are functional lipids formed by combining MCFAs and LCFAs, including MLM, MML, LMM, LML, MLL, and LLM (where L stands for LCFA and M stands for MCFA), are more digestible and absorbable [36]. The safety and functionality of MLCT products, a new healthy edible oil alternative to traditional oil, have gained approval in Europe, the United States, and Japan. From a health benefits perspective, MLCT not only offers nutritional advantages due to the essential fatty acids it contains but more significantly, it exhibits beneficial effects in combating obesity and enhancing insulin sensitivity. Long-term administration of MLCT, compared with LCT, can reduce the body weight of mice and inhibit fat accumulation. Terada et al [37] discovered that dietary intake of MLCT for six weeks resulted in increased adiponectin levels and improved insulin resistance in high-fat diet-induced obese rats. In addition, much more than the above beneficial effects, MLCT was also used as healthy cooking oil, energy bar, butter fat, margarine and shortening, beverage, and deep-frying oil [38]. Therefore, considering the composition of TGs in adipose tissue, a high-quality lipid is significantly enriched in yellow fat in aged cattle. These results demonstrate that yellow fat would be a promising food raw. For example, oil extracted from yellow fat is used as an alternative to edible vegetable oils used in cakes, pastries, biscuits, fermented sausages, and deep-frying oil. However, the relationship between the structural characteristics and digestion and absorption behavior of MLCT has not been fully understood due to a lack of systematic studies. Therefore, further in-depth, scientifically designed studies are needed to explore the digestive processes and characteristics of different MLCTs under physiological conditions. In the future, the potential value of yellow fat will be further explored to promote its utilization and improve its economic value.

Figure 5B shows the saturation levels at the three sites of upregulated and downregulated TG molecules in SDLs. In the SAT of 10 M cattle, the sn-1 and sn-3 sites of the TGs predominantly displayed SFAs, with no PUFAs detected. Conversely, in the SAT of 34 M cattle, the sn-1 and sn-2 sites of TG were primarily composed of SFAs, while the sn-3 site exhibited a higher proportion of monounsaturated fatty acids and PUFAs, such as DPA, linoleic acid, and α-linolenic acid. Examples of these TGs include TG (8:0_16:0_18:3), TG (10:0_15:0_18:2), TG (10:0_16:0_18:3), TG (10:0_16:0_18:2), TG (10:0_15:0_22:5), TG (16:0_18:2_18:3), and TG (17:1_18:1_18:3). This finding is consistent with that of Xiong et al [7], who reported high levels of PUFAs at the sn-3 position of TGs in the SAT of grazing cattle. Furthermore, based on our findings, PC fatty acids are predominantly saturated at the sn-1 site. In contrast, the sn-2 sites of PC contained a high proportion of PUFAs such as PC (18:2_20:4), PC (18:3_20:4), PC (18:1_20:4), PC (18:0_20:4), PC (20:4_20:4), and PC (20:3_20:3) (Figures 5C). In ruminants, PUFAs are preferentially deposited in phospholipids. Their primary roles in metabolism and physiology have helped elucidate their potential beneficial effects. In general, the consumption of PUFA-rich meat is beneficial to human health.

#### Metabolic pathway analysis

**Figure 6:**
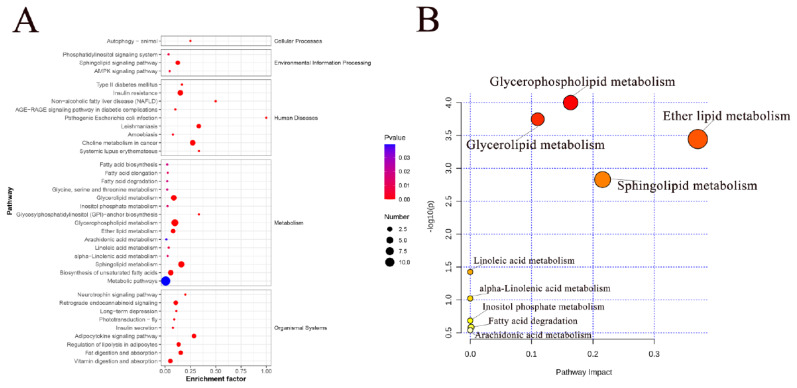
Metabolic pathway analysis of differential lipids. (A) Lipid metabolic pathway analysis of the identified differentially expressed lipid species. (B) Key metabolic pathways involved in lipid changes. The abscissa value and bubble size represent the degree of impact, with larger values indicating higher degrees of enrichment. The ordinate value and bubble color represented the enrichment analysis’s p-value; the darker the bubble color, the smaller the p-value, and the more significant the degree of enrichment.

To investigate the differences in the lipid metabolic pathways of SAT in Mongolian cattle, we performed a KEGG classification of 37 pathways related to 172 SDLs (Figure 6A). Using the MetaboAnalyst 5.0 platform (https://www.metaboanalyst.ca/), we conducted a topology analysis of these lipids and calculated p-values and pathway influences. Significance was attributed to correlation pathways with a p-value<0.05 and an effect value>0.1 (Figure 6B). Ten metabolic pathways associated with lipid changes were identified: GP metabolism, GL metabolism, sphingolipid metabolism, fatty acid degradation, ether lipid metabolism, inositol phosphate metabolism, arachidonic acid metabolism, linoleic acid metabolism, alpha-linolenic acid metabolism, and phosphatidylinositol signaling. Notably, the GP and GL pathways exhibited significant differences between the 10 and 34 M cattle groups, followed by ether lipid and sphingolipid metabolism. Differential lipids enriched in four significantly different metabolic pathways were presented in Supplement 5.

GPs are the major structural lipids in eukaryotic cell membranes that regulate various cellular metabolic processes. Moreover, phospholipases play crucial roles in phospholipid metabolism and cell signaling. GP metabolism primarily regulates PC, PS, and LPC, with PC and LPC being the predominant species. LPC levels received particular attention among the GPs of the SDLs identified in the 10 and 34 M groups, which exhibited the most significant changes. The ratio of LPC (20:3)/PC (20:3_20:3) increased from 1:7.30 to 1:0.89 between the 10 M and 34 M groups. This suggests that the lipid transformation of PC and LPC progressed via GP metabolism. Specifically, LPC is generated by the cleavage of PC molecules by phospholipase A2. Additionally, LPC and acyl-coenzyme A can form PC in the presence of lysophospholipid acyltransferase. Furthermore, DGs, which serve as raw materials for synthesizing PC, PE, PE-O, and PC-O, showed an upward trend in the SAT of 34 M cattle. The decrease in DG content in the 10 M group may be due to hydrolysis by various lipases [39]. Additionally, certain DG species may participate in the synthesis of PE and PC, which can occur either through corresponding phosphotransferases or CDP-DAGm acting as a lipid anchor [40].

## CONCLUSION

In natural grazing Mongolian cattle, the developmental stage exerted a significant impact on lipid composition and metabolism (Supplement 6). We found that the SAT from 34 M cattle exhibited more yellowness than from 10 M cattle. The expression of mitochondrial biogenesis-specific marker genes, thermogenic genes, and beige adipocyte marker genes was upregulated in the SAT of 34 M cattle. The browning of lipids increases with age, potentially affecting lipid composition, fat color, and lipid metabolism. Correlation analysis showed that *UCP2* may be involved in SAT browning in Mongolian cattle. Lipidomic analysis revealed 172 SDLs selected from 553 lipids in the 10 and 34 M age groups. These SDLs contained a high proportion of GLs and GPs. The proportion of phospholipids changed inversely compared with that of neutral lipids during development. In addition, many functional lipids, such as MLCT, FAHFA, and vitamin D, were enriched in yellow fat at 34 M. Pathway analysis indicated that the most significant metabolic pathways were GP and GL metabolism, followed by ether lipid and sphingolipid metabolism. This study offers new theoretical insights into the metabolic mechanisms of yellow fat formation during the growth of Mongolian cattle, and serves as a reference for the comprehensive utilization of yellow fat, enhancing its added value, and contributing to the meat industry chain.

## Figures and Tables

**Table 1:** Correlation analysis of *UCP2* and brown/beige adipocyte-related adaptive thermogenesis genes^1)^

Genes	*UCP2*	p-values
*UCP1*	0.986**	0.002
*PGC1α*	−0.913*	0.001
*PRDM16*	0.815*	0.048
*Cox8b*	0.776	0.069
*Cidea*	0.975**	0.001
*DIO2*	0.993**	<0.001

1) The correlation coefficient >0.8 or <−0.8 indicated a strong correlation.

* p<0.05,

** p<0.01.

*UCP1*, Uncoupling protein 1; *UCP2*, Uncoupling protein 2; *PGC1α*, Peroxisome proliferators-activated receptor gamma coactivator alpha; *PRDM16*, PR domain-containing; *Cox8b*, cytochrome c oxidase subunit 8B; *Cidea*, Cell death-inducing DFFA-like effector A; *DIO2*, Deiodinase, Iodothyronine, Type II.

## SUPPLEMENTARY MATERIAL

**Supplement 1.** The summary table of comparative literature findings

**Supplement 2.** The primer sequences for quantitative real-time polymerase chain reaction

**Supplement 3.** Significantly different lipids of Mongolian cattle between 10 (10 M) and 34 months old (34 M)

**Supplement 4.** Five significantly different lipids (SDLs) with the highest VIP values (sorted from high to low) between 10 (10 M) and 34-month-old (34 M) Mongolian cattle.

**Supplement 5.** Metabolic pathway with significant enrichment of differential lipids

**Supplement 6.** Graphical abstract
