# Supplementary material for: Change in adipose tissue characteristics and lipid metabolism in natural grazing Mongolian cattle with age
Source: Anim Biosci. 2025 Feb 27;38(8):1784–97. doi: 10.5713/ab.24.0706 (PMC12229929; doi:10.5713/ab.24.0706)
Supplement: Supplementary file 1 [file ab-24-0706-Supplementary-1.pdf]

Supplement 1. The summary table of comparative literature findings

Reference	items	Comparison types	Animals	Tissue types	comparison results
[11]	Color measures	Grass silage, whole crop wheat silage, ad libitum concentrates, maize silage, and alkalage.	Crossbred steers	Subcutaneous fat	The b^* value of the subcutaneous fat of cattle fed a green grass diet was higher than that of other groups.
[12]	Color measures	Summer and winter	Cattle	Longissimus thoracic	L^*, a^*, and b^* values increased significantly in winter, which were higher than in summer.
[14]	Color measures	Different ages	Maremmana cattle	Muscle	With the increase in age, L^*, a^*, and b^* increase.
[16]	Color measures	Different ages	Danish cull cows	Adipose tissue	As the cow advances in age, the carcass color tends to become darker yellow.
[23]	Expression of genes	Roughage diet and high-energy diet	Japanese Black steers.	Subcutaneous fat	UCP1, Dio2, Cox1, Cox8b, and PRDM16 expression in the subcutaneous WAT was significantly higher in the concentrate diet group than in the roughage diet group.
[24]	Expression of genes	High energy diet+ high temperature, low energy die+high temperature, and low energy die+low temperature	Holstein bull calves	iWAT	Cold exposure rather than high energy diet-induced transcript elevations of Cold exposure rather than high energy diet-induced transcript elevations of Cited1, Tbx1, Cidea, UCP1, NRF1, NRF2, TFAM, PGC1α ($p < 0.05$).

[25]	Expression of genes	Sedentarism and long-term treadmill training	Aged obese mice	iWAT	iWAT of trained animals showed an increment in the expression of PGC1α, TFAM, NRF1, UCP1,CD137, and Tbx1.
[6]	Metabolism	Different fat parts	Huaxi cattle	Adipose tissues	The TG content in SAT was significantly higher than those of VAT and AAT, while the relative contents of DG and LPC were significantly lower than those of VAT and AAT. There was no significant difference in the contents of 12 lipids classes between VAT and AAT, implying VAT and AAT were more similar.
[7]	Metabolism	Different feeding patterns	Yak (Bos grunniens)	Subcutaneous fat	Compared with the GF group, the percentage of PEs, SMs, and TGs in the SF group was higher, whereas the rate of DGs was lower.
[8]	Metabolism	Different ages	Lilu yellow cattle	Subcutaneous fat	Energy metabolism was active from 12 to 24 months but inhibited at 30 months. In addition, 18 months is a key age for alterations in metabolism.
[9]	Metabolism	Cold exposure	Mice	Adipose tissues	Long-chain and odd-numbered acyl chains in TAGs are increased in response to cold.

iWAT, Inguinal fat; SAT, subcutaneous adipose tissues; VAT, visceral adipose tissues; AAT, abdominal adipose tissues; SF, Stall feeding; GF, graze feeding.
