# Supplementary material for: Change in adipose tissue characteristics and lipid metabolism in natural grazing Mongolian cattle with age
Source: Anim Biosci. 2025 Feb 27;38(8):1784–97. doi: 10.5713/ab.24.0706 (PMC12229929; doi:10.5713/ab.24.0706)
Supplement: Supplementary file 2 [file ab-24-0706-Supplementary-2.pdf]

**Supplement 2.** The primer sequences for quantitative real-time polymerase chain reaction

| Gene | GenBank ID | Primer sequence（5’→3’） | Length（bp） |
|---|---|---|---|
| UCP1 | NM_001166528.1 | F: GCACAACGGTTCTGTCCTCTCC<br>R: ACGGTCCTTCCCTAGTGAGCATC | 120 |
| UCP2 | NM_001033611.2 | F: TGCCATTGTCAACTGTGCTGAG<br>R: AAGGGAGGTCGTCTGTCATTAGG | 89 |
| PRDM16 | XM_024976786.1 | F: CAAGGATTGCGAGCGGATGTTC<br>R: CTGGTGGCGGATGAGGTTGG | 136 |
| PGC1α | XM_024993058.1 | F: GACACCGCACACACCGAAATTC<br>R: GGATTCCCGCTTCTCATACTCTCTG | 149 |
| DIO2 | NM_001010992.7 | F: AGCCTTTGAACGTGTGTGCATC<br>R: TGAAATTCTTCTCCAGCCAACGC | 113 |
| Cidea | NM_001083449.1 | F: TGGTGGACACAGAGGAGTTCTTCC<br>R: ACGTTGAGGCAGCCAATGACATC | 193 |
| Cox8b | NM_001114517.2 | F: TCTGTGACGTTCCTCAGCTTC<br>R: TGCTGCTGAGCTCTTCTTGTAG | 81 |

| | | | |
|---|---|---|---|
| CD137 | NM_001035336.2 | F: ACAGTTTCTCCAGCACAAGTGG<br>R: TTCACACTCTGCATTGCTGGTG | 110 |
| Tmem26 | NM_001103163.3 | F: TTCCTTGTTGTGCGGCTCATAC<br>R: AAGGAAGCGCGTACATCCAAG | 143 |
| Tbx1 | XM_024977887.2 | F: AGTGAAGCTGTTTGGCATGGAC<br>R: TACCGGTAGCGCTTGTCATCC | 84 |
| Cited1 | NM_174518.1 | F: ATGTCGCTGGTGGTGGAACTG<br>R: GGAAAGTCTGCCGTGAAGTCAAAC | 95 |
| NRF1 | NM_001098002.2 | F: TGATGGCACTGTCTCGCTTATCC<br>R: GTGACTGTGGTTGGCAATTCTGAAG | 87 |
| NRF2 | NM_001011678.2 | F: GCTCTCCATATCCCATTCCCTGTAG<br>R: CTCGTTGAATTGCTCCTTGGACATC | 90 |
| TFAM | NM_001034016.2 | F: GTGGTTATCCAAAGAAGCCCATGAC<br>R: TGAATCAGGAAGTTCCCTCCATAGC | 143 |
| ND1 | NC_006853.1 | F: GACCATTTGCCCTCTTCTTCATAGC<br>R: GGTATGTGTGGATTGTGGGATGTTC | 103 |

| | | | |
|---|---|---|---|
| GAPDH | NM_001034034.2 | F: AACGGCACAGTCAAGGCAGAG<br>R: CGCTCCTGGAAGATGGTGATGG | 71 |

UCP1, Uncoupling protein 1; UCP2, Uncoupling protein 2; PRDM16, PR domain-containing; PGC1α, Peroxisome proliferators-activated receptor gamma coactivator alpha; DIO2, Deiodinase, Iodothyronine, Type II; Cidea, Cell death-inducing DFFA-like effector A; Cox8b, cytochrome c oxidase subunit 8B; CD137, cluster of differentiation 137; Tmem26, transmembrane protein 26; Tbx1, T-box transcription factor 1; Cited1, Cbp/p300 interacting transactivator with Glu/Asp rich carboxy-terminal domain 1; NRF1, nuclear respiratory factor 1; NRF2, nuclear respiratory factor 2; TFAM, mitochondrial transcription factor A; ND1, NADH dehydrogenase subunit 1; GAPDH, glyceraldehyde-3-phosphate dehydrogenase.
