# Supplementary material for: Change in adipose tissue characteristics and lipid metabolism in natural grazing Mongolian cattle with age
Source: Anim Biosci. 2025 Feb 27;38(8):1784–97. doi: 10.5713/ab.24.0706 (PMC12229929; doi:10.5713/ab.24.0706)
Supplement: Supplementary file 3 [file ab-24-0706-Supplementary-3.pdf]

Supplement 3. Significantly different lipids of Mongolian cattle between 10 (10 M) and 34 months old (34 M)

Name	Rt[1)]	Mz[2)]	VIP[3)]	*p*-value[4)]	FC[5)]	Trends[6)]
TG 16:0_18:0_28:0	9.217117	1020.973	1.302232	0.007597709	0.385323	down
TG 18:1_22:0_23:0	9.2662	1032.979	1.188902	0.021030406	0.390682	down
TG 18:0_18:1_28:0	9.340625	1046.995	1.380777	0.015531365	0.293107	down
TG 18:1_18:1_30:0	9.32645	1073.011	1.301684	0.014455551	0.325879	down
TG 18:0_18:1_30:0	9.440992	1075.025	1.372697	0.015043845	0.313485	down
TG 18:1_18:1_32:0	9.418742	1101.041	1.400476	0.00407359	0.348629	down
TG 8:0_12:0_16:0	7.041267	656.5792	2.036376	0.004488981	4.776937	up
TG 8:0_14:0_15:0	7.166342	670.5944	1.830354	0.003158486	3.864631	up
TG 10:0_12:0_16:1	7.051108	682.5947	2.070821	0.004122698	5.053774	up
TG 8:0_14:0_16:0	7.311958	684.6113	1.992248	0.00896816	4.14299	up
TG 10:0_10:0_18:0	7.30525	689.565	1.71567	0.002837779	3.53163	up
TG 10:0_12:0_17:1	7.179092	696.6097	1.891909	0.005550418	3.927324	up
TG 8:0_15:0_16:0	7.4082	698.626	1.786849	0.010679977	3.276553	up
TG 13:0_13:0_13:0	7.40865	703.5796	1.462252	0.008986668	2.511211	up
TG 10:0_14:0_16:3	6.847067	706.5938	1.58979	0.007113293	2.955903	up
TG 8:0_16:0_16:1	7.314117	710.6266	2.123049	0.008286612	4.839743	up
TG 10:0_14:0_16:0	7.556867	712.6421	1.75247	0.012971181	3.17737	up
TG 12:0_12:0_16:0	7.555608	717.5955	1.439834	0.005215512	2.519211	up
TG 10:0_14:1_17:1	7.199275	722.6244	1.721241	0.010621177	3.098141	up
TG 8:0_15:0_18:1	7.419758	724.6416	1.86763	0.017489471	3.170824	up

TG 8:0_16:0_17:1	7.669333	724.6407	1.613965	0.003911722	3.021161	up
TG 10:0_15:0_16:0	7.639775	726.6569	1.649058	0.02628742	2.509696	up
TG 13:0_14:0_14:0	7.640567	731.6103	1.288094	0.01810737	2.020253	up
TG 8:0_16:0_18:3	7.094367	734.6246	1.881445	0.011448878	3.499731	up
TG 8:0_16:1_18:1	7.315667	736.6418	1.938562	0.020947836	3.166476	up
TG 8:0_16:0_18:1	7.545458	738.6573	1.907065	0.022080846	3.206133	up
TG 14:0_14:0_14:1	7.544517	743.6113	1.555517	0.013136951	2.655836	up
TG 10:0_15:0_18:2	7.439833	750.6565	1.713667	0.039166087	2.297714	up
TG 10:0_16:0_17:1	7.887708	752.6712	1.327734	0.011675075	2.176728	up
TG 10:0_14:0_19:2	7.43495	755.6111	1.477892	0.038473496	2.078639	up
TG 10:0_16:0_18:3	7.345617	762.6568	1.88123	0.039443074	2.427477	up
TG 10:0_16:0_18:1	7.767742	766.6883	1.555808	0.032613515	2.369153	up
TG 10:0_16:0_18:2	7.549542	769.626	1.484201	0.041321047	2.098235	up
TG 14:0_16:0_16:0	8.200208	796.7346	1.102707	0.028012513	0.464064	down
TG 10:0_15:0_22:5	7.7429	800.6708	1.691069	3.45E-06	2.803684	up
TG 15:0_16:0_16:0	8.260533	815.704	1.310212	0.042132989	0.307852	down
TG 14:0_16:0_18:0	8.391567	824.7648	1.21329	0.025092411	0.391792	down
TG 16:0_16:0_18:4	7.8032	849.6896	1.726139	0.042777907	2.441824	up
TG 16:0_16:0_18:0	8.58465	852.7928	1.222567	0.013001213	0.384992	down
TG 16:0_16:0_18:1	8.369267	855.7342	1.178925	0.02983255	0.411739	down
TG 15:0_17:0_19:1	5.779133	864.7961	1.39827	0.011891597	0.438663	down
TG 16:0_18:2_18:3	7.83195	870.7504	1.491104	0.046515232	2.065629	up

TG 17:1_18:1_18:3	7.897125	884.7655	1.264937	0.008652248	2.062913	up
TG 17:0_17:1_19:0	5.77935	892.8271	1.420514	0.005124566	0.450234	down
TG 17:0_18:0_18:1	8.07085	892.8248	1.398499	0.021008462	0.262972	down
TG 11:0_20:1_22:0	8.6112	897.7786	1.136815	0.014768483	0.47434	down
LPC 36:2	6.414525	816.6045	1.791514	0.000563536	0.220201	down
LPC 20:3-SN1	2.116083	546.354	1.087859	0.028826958	2.01478	up
LPC 30:2-SN2	5.531767	688.5229	1.205887	0.011195333	0.420292	down
LPC 30:1-SN2	5.91775	690.5392	1.444714	0.003948993	0.33965	down
LPC 30:0-SN2	6.026125	692.5538	1.476295	0.004126201	0.323504	down
LPC 32:4-SN2	5.167858	712.523	1.376934	8.17E-05	0.438982	down
LPC 32:3-SN2	5.541933	714.5397	1.94116	0.000200478	0.198583	down
LPC 32:2-SN2	5.907167	716.554	1.552197	0.00235826	0.301962	down
LPC 32:1-SN2	6.356283	718.5699	1.767671	0.000958743	0.216732	down
LPC 32:0-SN2	6.393033	720.5834	1.566553	0.001608776	0.298426	down
LPC 34:3-SN2	6.031617	742.5695	2.299202	6.45E-05	0.107961	down
LPC 34:2-SN2	6.084417	744.5854	2.225012	0.000350418	0.101918	down
LPC 34:1-SN2	6.391217	746.6039	1.989752	0.001418804	0.137202	down
LPC 36:7-SN2	5.21735	762.5385	1.362557	0.005015329	0.35899	down
LPC 36:6-SN2	5.399375	764.5539	1.489046	0.000166937	0.365934	down
LPC 36:5-SN2	5.96025	766.5731	1.194296	0.001065518	0.497355	down
LPC 36:3-SN2	6.153625	770.5984	1.334858	0.006568756	0.361485	down
LPC 36:2-SN2	6.431383	772.6154	2.073004	0.000857663	0.118089	down

LPC 36:1-SN2	6.735908	774.642	1.35291	0.003498276	0.371112	down
LPC 38:6-SN2	5.92165	792.5867	1.487236	0.000636733	0.345241	down
DG 14:0_15:0	6.16505	544.4905	1.587204	0.046246536	2.084538	up
DG 14:0_16:0	6.411717	558.5072	2.072008	0.018751335	3.759583	up
DG 15:0_16:0	6.52705	572.5221	1.840619	0.011974845	3.409553	up
DG 14:0_18:1	6.404	584.5227	1.802618	0.043835486	2.3196	up
DG 16:0_16:0	6.751692	586.538	1.779574	0.002999231	3.727681	up
DG 15:0_18:1	6.568458	598.5375	1.609245	0.027464738	2.41847	up
DG 16:0_17:1	6.842067	598.5367	1.721896	0.006200864	3.266815	up
DG 15:0_18:0	6.856717	600.5535	1.8333	0.003566247	3.942728	up
DG 16:0_18:1	6.743217	612.5541	1.831812	0.029487109	2.768797	up
DG 16:0_18:0	7.056867	614.5696	1.662083	4.47E-05	3.310583	up
DG 17:0_18:1	6.869008	626.569	1.623119	0.031780222	2.328421	up
DG 17:0_18:0	7.164492	628.5843	1.774371	0.000550362	4.101038	up
DG 18:0_18:1	7.048475	640.5849	1.453234	0.028566213	2.150837	up
DG 18:0_18:0	7.324758	642.6004	1.163442	0.013945957	2.125617	up
DG 16:0_21:0	7.440117	656.6137	1.466588	0.002962262	2.907185	up
DG 16:0_22:0	7.588233	670.6297	1.520082	0.002962203	3.187176	up
PC O-14:1_18:2	5.55625	758.5273	1.593932	9.88E-05	0.345282	down
PC O-14:1_18:1	5.9216	760.542	1.588378	0.00011518	0.317439	down
PC O-15:1_18:1	6.099183	774.5577	1.480298	0.000976208	0.346002	down
PC O-18:1_16:0	6.392658	790.59	1.784339	0.001588489	0.20665	down

PC O-16:1_20:5	5.62205	808.5421	1.237637	0.001292073	0.472248	down
PC O-18:2_18:1	6.148533	814.5879	1.853558	0.000415661	0.220249	down
PC O-16:1_20:2	6.38395	814.5892	1.634182	2.62E-05	0.327405	down
PC O-29:1	5.7078	676.5222	1.519533	0.001381283	0.319895	down
PC O-31:1	6.09615	704.555	1.36833	0.008627613	0.36058	down
PC O-33:4	5.424433	726.539	1.471085	0.002704359	0.336497	down
PC O-33:3	5.7568	728.5544	1.691086	0.000480865	0.266111	down
PC O-33:2	6.086442	730.5707	1.653004	0.000197087	0.290598	down
PC O-33:1	6.465017	732.5837	1.630059	0.001777967	0.264147	down
PC O-35:3	6.142883	756.5842	1.901289	0.000120528	0.197824	down
PC O-35:2	6.443558	758.6011	1.918672	0.000156501	0.205121	down
PC O-37:7	5.466883	776.5555	1.430016	0.014419684	0.271066	down
PC O-37:6	5.635083	778.5693	1.134001	0.020753616	0.478917	down
PC O-37:5	6.038308	780.586	1.313216	0.00188939	0.419552	down
PC 16:0_15:1	6.260767	718.5351	1.345975	0.00707383	2.286261	up
PC 12:0_19:0	5.909467	720.5503	1.450304	0.004572822	0.330817	down
PC 15:0_18:2	5.6386	744.5493	1.266218	0.002551683	0.456609	down
PC 15:1_18:1	6.339533	744.5819	2.220752	0.00014252	0.116735	down
PC 14:0_19:0	6.277375	748.5809	1.390981	0.005558672	0.357614	down
PC 18:3_20:4	4.993883	804.5487	1.801399	8.28E-06	0.264353	down
PC 18:2_20:4	5.407917	806.5655	1.617352	0.000119219	0.315602	down
PC 18:1_20:4	5.757733	808.5813	1.064369	0.024303336	0.479295	down

PC 18:0_20:4	6.12765	810.5973	1.214392	0.004811383	0.451991	down
PC 20:4_20:4	5.220383	830.5646	1.411328	0.004491253	0.334499	down
PC 20:3_20:3	6.000117	834.5948	1.435892	0.022369606	0.245048	down
TG O-10:0_8:0_8:0	5.587983	507.3997	1.723789	0.021126138	2.763358	up
TG O-8:0_10:0_10:0	6.031433	535.4302	1.619181	0.01339178	2.79414	up
TG O-8:0_8:0_14:1	6.0453	561.4463	1.658301	0.022520412	2.563051	up
TG O-10:0_10:0_10:0	6.411633	563.4613	2.100255	0.004173302	5.557843	up
TG O-16:2_8:0_8:0	6.075233	587.4607	1.676418	0.032062506	2.389447	up
TG O-8:0_8:0_16:1	6.401658	589.477	1.870438	0.010625357	3.654361	up
TG O-8:0_12:0_12:0	6.74945	591.4923	1.621063	0.000129482	3.299811	up
TG O-18:3_8:0_8:0	6.179117	613.4753	1.549988	0.00573242	2.749925	up
TG O-18:2_8:0_8:0	6.451633	615.4927	1.738907	0.010206137	3.239109	up
TG O-10:0_12:0_12:0	7.049142	619.5235	1.55321	0.000365109	2.924729	up
TG O-16:3_10:0_10:0	6.4863	641.5063	1.464525	0.00763398	2.540723	up
TG O-16:1_10:0_10:0	7.047717	645.5391	1.472257	0.006703965	2.674954	up
TG O-10:0_16:0_16:0	8.079942	726.69	1.217747	0.02235079	0.351324	down
TG O-16:0_14:0_14:0	8.277533	754.7222	1.379494	0.026563274	0.248632	down
TG O-10:0_18:1_18:1	8.0763	778.7207	1.720137	0.003761302	0.216548	down
TG O-14:1_16:0_16:0	8.267675	780.7382	1.395303	0.035817313	0.224043	down
PE O-14:1_18:2	5.706817	670.4757	1.55502	4.01E-05	0.359407	down
PE O-24:6_18:2	6.146633	800.5728	1.725037	3.43E-05	0.3105	down
PE O-15:1_18:2	5.894967	686.5075	1.810347	0.000132777	0.237314	down

PE O-16:1_18:2	6.148308	700.5239	2.21012	0.000102689	0.119966	down
PE O-16:1_18:1	6.444633	702.5397	1.474799	0.002465004	0.333249	down
PE O-17:1_18:2	6.261133	714.539	2.003212	0.000115414	0.176752	down
PE O-18:1_18:2	6.486475	728.5546	1.651155	0.000951217	0.269557	down
PE O-18:1_20:3	6.547917	754.5703	1.720943	1.67E-05	2.564603	up
SHexCer 33:1;3O	6.269717	780.5095	1.296279	0.001271153	0.43971	down
SHexCer 34:2;3O	6.0532	792.5021	1.158225	0.020360015	2.089042	up
SHexCer 35:1;2O	6.274633	792.5661	1.151751	0.011201864	0.475792	down
SHexCer 34:1;3O	6.485608	794.5257	1.234438	0.001807604	0.460483	down
SHexCer 36:2;3O	6.556083	820.5399	1.419238	0.000169731	2.053462	up
SHexCer 37:0;3O	5.983083	838.5882	1.29079	0.000862613	0.450528	down
SHexCer 40:1;3O	6.011617	878.5828	1.664859	0.009443878	0.175599	down
FA 22:6	2.712925	327.2308	1.271798	0.021714158	0.331054	down
FA 22:5	2.956283	329.2464	1.461927	0.001141498	0.449582	down
FA 22:0	5.767242	339.324	1.348377	0.002947528	2.409986	up
FA 28:0	6.989367	423.4166	1.377589	0.000532845	2.448413	up
SM 8:0;2O/25:0	5.916967	691.5432	1.372451	0.004623019	0.37643	down
SM 8:0;2O/28:0	6.411633	733.6163	1.546149	6.19E-05	0.392898	down
SM 49:0;2O	8.669217	937.8102	1.151546	0.026693793	0.417819	down
ST 29:1;O;Hex;FA 16:1	7.12715	871.6595	1.547999	0.001376515	2.5874	up
ST 29:1;O;Hex;FA 17:1	7.27865	885.6754	2.740212	0.000105201	16.53147	up
ST 29:1;O;Hex;FA 17:0	7.521233	887.6904	2.235803	0.000191151	6.611851	up

ST 29:1;O;Hex;FA 26:3	7.026317	993.7514	1.655451	0.029668076	2.329273	up
ST 29:1;O;Hex;FA 26:2	7.1596	995.7659	1.625496	0.02995353	2.531683	up
Cer 18:1;2O/16:0	6.203108	538.5169	1.622618	0.000100777	0.319101	down
Cer 18:1;2O/18:1	6.220158	564.5308	1.659012	6.70E-06	0.360762	down
Cer 18:1;2O/18:0	6.568667	566.5474	1.765315	3.19E-05	0.288338	down
Cer 18:1;2O/23:0	7.3163	636.6252	1.222989	0.002366776	0.462328	down
Cer 18:1;2O/24:0	7.446175	650.6406	1.125828	0.006784592	0.491728	down
MGDG O-8:0_26:0	7.34725	767.6111	1.504016	0.033049094	2.20247	up
MGDG O-9:0_28:1	7.5878	807.6436	2.019526	0.010190033	0.321675	down
MGDG O-12:0_28:0	7.9833	851.7054	1.95059	0.0265924	3.385568	up
MGDG O-14:1_28:0	7.992183	877.7207	1.864345	0.019949106	3.517853	up
DG O-18:1_20:4	6.910833	646.5729	1.193166	0.006250667	2.039034	up
DG O-18:0_22:5	6.950917	674.6029	1.401897	3.67E-05	2.331794	up
FAHFA 18:0/20:2	4.443583	589.5127	1.245307	0.001158174	2.058312	up
LPC O-16:1	2.524183	480.3426	1.555958	0.001003396	0.324367	down
LPC O-18:0	3.349042	510.3901	1.307301	0.000547022	0.458729	down
SMGDG O-22:0_28:0	7.137067	1047.798	1.731051	0.024118458	2.911553	up
PS 18:0_20:3	6.084258	812.5392	1.351946	0.000299742	2.162327	up
PC 18:0_18:1;3O	6.115575	880.5994	1.532465	0.000367721	0.355461	down
25-hydroxycholecalciferol	3.625467	401.3403	1.812216	0.011236121	3.153098	up

[1)] Rt: the chromatographic retention time of the substance.

[2)] mz: the mass-to-charge ratio of characteristic ions in a substance.

[3)] VIP: variable importance in projection.

[4)] *p*-Value: obtained from the t-test of the substance in this group comparison.

[5)] FC: fold change.

[6)]Trends: the expression trend of SDLs.
