# Supplementary material for: Change in adipose tissue characteristics and lipid metabolism in natural grazing Mongolian cattle with age
Source: Anim Biosci. 2025 Feb 27;38(8):1784–97. doi: 10.5713/ab.24.0706 (PMC12229929; doi:10.5713/ab.24.0706)
Supplement: Supplementary file 4 [file ab-24-0706-Supplementary-4.pdf]

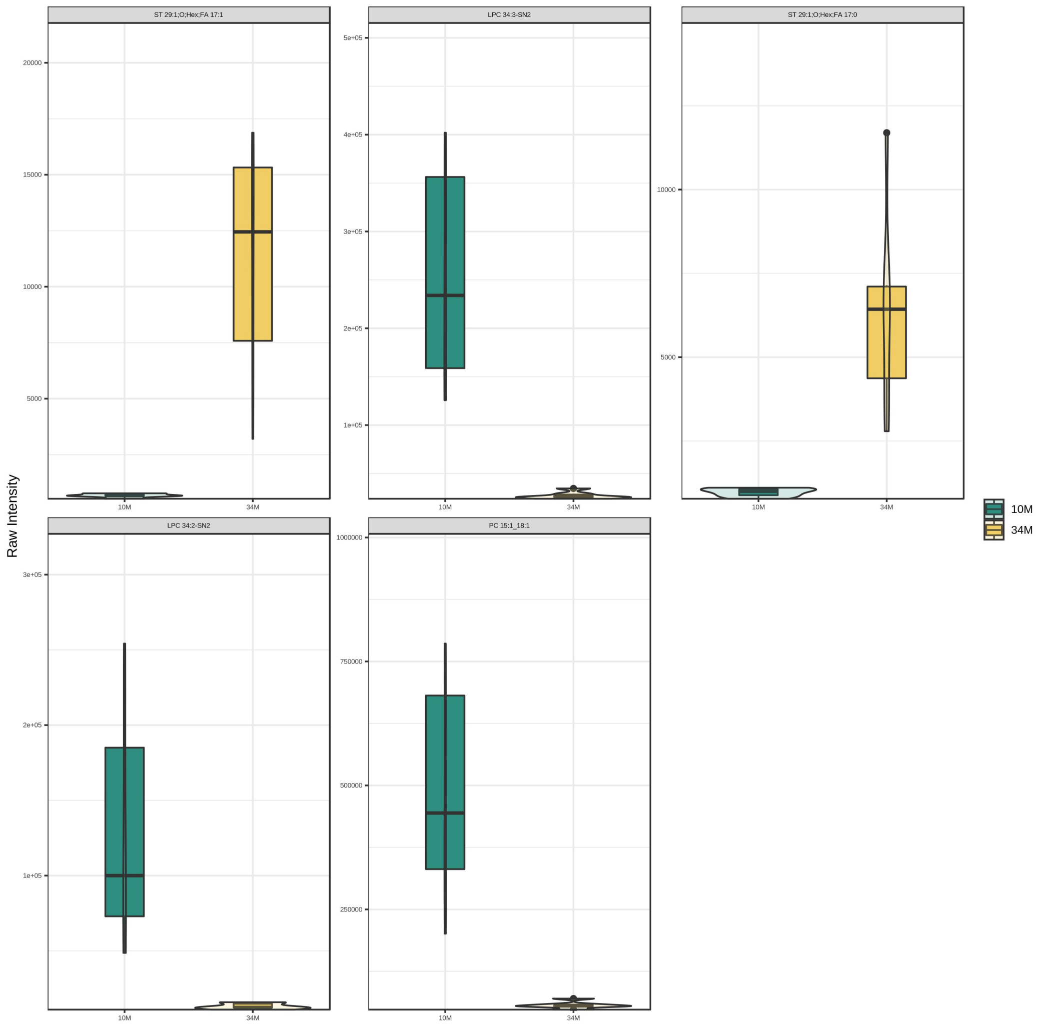

Supplement 4. Five significantly different lipids (SDLs) with the highest VIP values (sorted from high to low) between 10 (10 M) and 34-month-old (34 M) Mongolian cattle.
