# Supplementary material for: Change in adipose tissue characteristics and lipid metabolism in natural grazing Mongolian cattle with age
Source: Anim Biosci. 2025 Feb 27;38(8):1784–97. doi: 10.5713/ab.24.0706 (PMC12229929; doi:10.5713/ab.24.0706)
Supplement: Supplementary file 5 [file ab-24-0706-Supplementary-5.pdf]

Supplement 5. Metabolic pathway with significant enrichment of differential lipids

Pathway Name	Lipid[1)]	*p*-value[2)]	Impact value[3)]
Glycerophospholipid metabolism	LPC (C04230), DG (C00641), PC (C00157), PS (C02737)	1.001E-4	0.16365
Glycerolipids metabolism	TG (C00422), DG (C00641), FFA (C00162)	1.7923E-4	0.10997
Ether lipid metabolism	PC-O (C05212), PE-O (C04475), LPC-O (C04317)	3.5862E-4	0.37107
Sphingolipids metabolism	SHexCer (C06125), SM (C00550), Cer_NS (C00195)	0.0014814	0.21576

Note: triacylglycerol = TG, diacylglycerol = DG, free fatty acid = FFA, phosphatidylcholine = PC, lysophosphatidylcholine = LPC, Phosphatidylserine = PS, sphingomyelin = SM, Ceramide non-hydroxy fatty acid-sphingosine = Cer_NS, Sulfatide = SHexCer, Ether-linked phosphatidylcholine = PC-O, Ether-linked phosphatidylethanolamine = PE-O, Ether-linked lysophosphatidylcholine = LPC-O.

[1)] Lipid: types of lipids in the pathway.

[2)] *p*-value: the *p*-value calculated from the enrichment analysis.

[3)] Impact value: the pathway impact value calculated from pathway topology analysis.
