# Supplementary figures and images for: Change in adipose tissue characteristics and lipid metabolism in natural grazing Mongolian cattle with age
Source: Anim Biosci. 2025 Feb 27;38(8):1784–97. doi: 10.5713/ab.24.0706 (PMC12229929; doi:10.5713/ab.24.0706)

Supplement 6. Graphical abstract

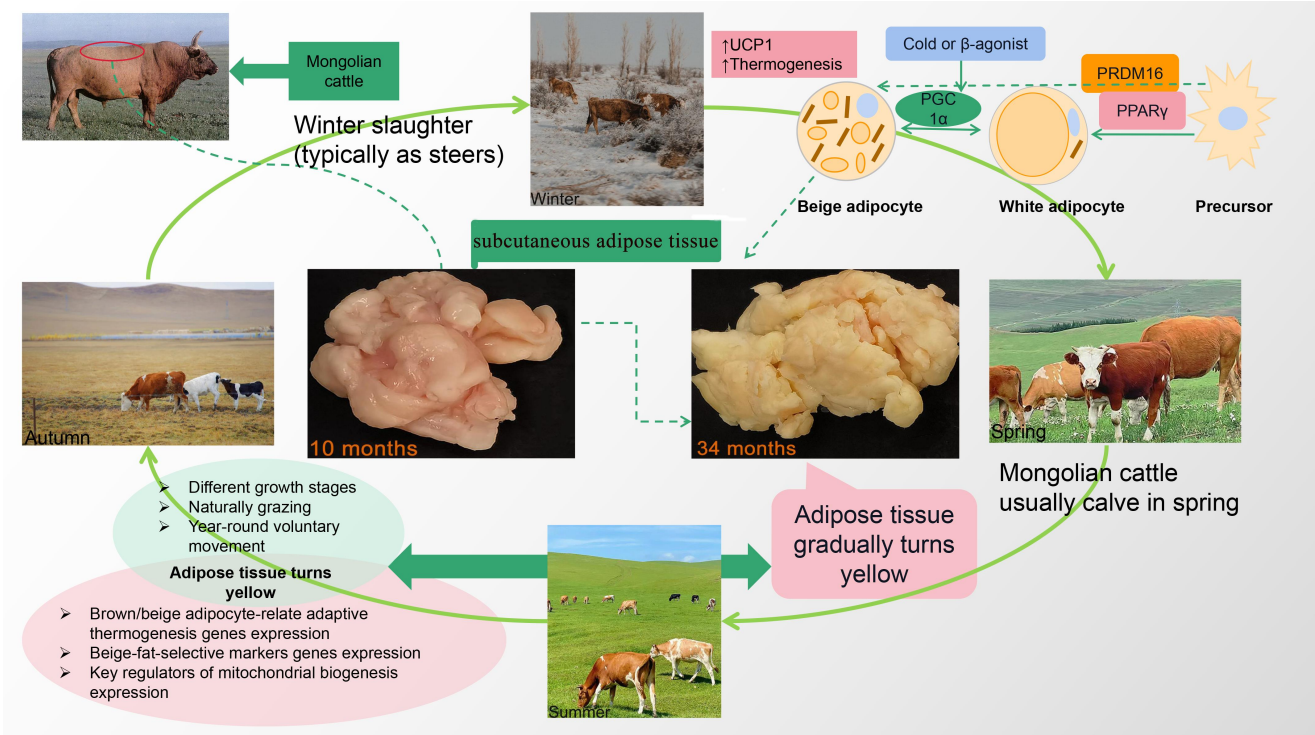

Supplement: Supplementary file 6 [file ab-24-0706-Supplementary-6.pdf]
